# The Impact of Resistance Training on Equilibrium Abilities and Quality of Life in Older Adults after SARS-CoV-2 Survival

**DOI:** 10.3390/jcm13102747

**Published:** 2024-05-07

**Authors:** Patrycja Bobowik, Jan Gajewski, Ida Wiszomirska, Agnieszka Maciejewska-Skrendo, Katarzyna Leźnicka, Katarzyna Kaczmarczyk

**Affiliations:** 1 Faculty of Rehabilitation, Józef Piłsudski University of Physical Education in Warsaw, Marymoncka 34, 00-968 Warsaw, Poland; ida.wiszomirska@awf.edu.pl (I.W.); katarzyna.kaczmarczyk@awf.edu.pl (K.K.); 2 Faculty of Physical Education, Józef Piłsudski University of Physical Education in Warsaw, Marymoncka 34, 00-968 Warsaw, Poland; jan.gajewski@awf.edu.pl; 3 Faculty of Physical Culture, Gdansk University of Physical Education and Sport, 80-336 Gdansk, Poland; agnieszka.maciejewska-skrendo@awf.gda.pl (A.M.-S.); katarzyna.leznicka@awf.gda.pl (K.L.); 4 Institute of Physical Culture Sciences, University of Szczecin, 70-453 Szczecin, Poland

**Keywords:** COVID-19, elderly, postural stability, resistance training, post-COVID-19 symptoms

## Abstract

**Background:** The scientific literature on COVID-19 and its long-term impacts on all-body systems and their treatments is still limited. The aim of the study was to create a safe protocol-based intervention to improve functional and equilibrium abilities in older adults impacted by COVID-19. **Methods:** This study used a sample of 46 people (intervention group: n = 26; control group: n = 20). Resistance training (RT) was held twice a week, with 60 min per session for 8 weeks. The postural stability and quality of life questionnaire (WHQOOL) was completed during pre- and post-testing. **Results:** The results indicated significant differences in overall stability index (OSI) with eyes open (EO), anterior–posterior stability index (APSI) EO, fall-risk index 6-2 (FRI6-2) values in males (*p* < 0.05), and APSI EO (*p* < 0.05) values in females compared to control groups, respectively. In the training, a significant improvement was reported in OSI EO and APSI EO (*p* < 0.05) female groups compared to baseline results and in FRI6-2 values in both gender groups (*p* < 0.01—men, *p* < 0.05—women). The effect of the intervention was recorded in the intervention group in the OSI EO (Z = −3.12, *p* < 0.01, R = 0.533) and FRI6-2 (Z = −2.06, *p* < 0.05, R = 0.354). Additionally, significantly different reactions between the groups were observed in the psychological domain (DOM2) (Z = 2.194, *p* < 0.028, R = 0.389), social relationship domain (DOM3) (Z = 2.051, *p* < 0.0403, R = 0.361), and in question 2 concerning general health (Z = 3.309, *p* < 0.0009, R = 0.535). **Conclusions:** The findings indicate that RT had a positive effect on older adults affected by COVID-19, led to a significant improvement in their postural stability, and had a significant impact on elements of psychological well-being and quality of life.

## 1. Introduction

In February 2020, the World Health Organization (WHO) named the condition caused by SARS-CoV-2 the coronavirus disease 2019 (COVID-19), and it became a topic of interest in the medical field as a major public health problem worldwide [1]. Post-COVID-19 conditions occur in individuals with a history of confirmed or probable SARS-CoV-2 infection when experiencing symptoms lasting at least 2 months, which initially occurred within 3 months of acute COVID-19 [2]. The evidence suggests that COVID-19 can cause lasting health consequences; however, long-term impacts on the all-body systems from COVID-19 infection are still not clear. A variety of persistent symptoms have been reported, including fatigue, headache, attention disorders, depression, hearing problems, gait instability, and dizziness [3,4]. It seems that SARS-CoV-2 can cause peripheral neuropathy and invade the neural pathways involved in body balance [3].

Despite the growing amount of scientific literature on COVID-19, studies that correlate audiovestibular symptoms to SARS-CoV-2 infection are still limited [5]. In previous studies, we demonstrated a significantly lower level of body balance abilities in older women after recovering from COVID-19 compared to age-matched healthy individuals [6]. Proper postural stability also requires the integration of the inner ear and nervous system [7]. Equilibrium disorders in post-COVID-19 survivors can be dependent on vascular damage. The inner ear structures are particularly susceptible to ischemia due to their characteristics of terminal vasculature and high-energy requirement [3,5]. Moreover, audiovestibular symptoms can present episodes of dizziness, which can lead to falls. Post-COVID-19 balance disorders may result from inflammation of the nervous tissue or ascending neural pathway impairments [4].

Long-term health consequences often result in limitations in daily functioning and affect quality of life (QOL). The negative effects of the COVID-19 pandemic on the mental health of the population are all too evident, especially in those at increased risk of infection, such as the elderly [8]. The long-term consequences of COVID-19, such as feelings of fatigue, deterioration of exercise tolerance, and reduced mood, negatively affect quality of life. Measuring QOL, especially for older people affected by COVID-19, is particularly important. The World Health Organization Quality of Life Group (1998) defined QoL as an “individuals’ perception of their position in life in the context of the culture and value systems in which they live and in relation to their goals, expectations, standards and concerns” [9].

The WHOQOL-BREF scale is based on the WHOQOL-100 questionnaire, which was developed on behalf of the World Health Organization as a universal survey tool to assess quality of life. It covered six domains: physical health, mental health, aspects of functioning, self-efficacy, social relationships, environment and religion, and global quality of life and self-rated health. Its shortened version, the WHOQOL-BREF scale, is intended to serve mainly clinical purposes [10]. The scale analyzes the four domains, as well as the global quality of life and self-rated health. It is worth mentioning that the tool was already adapted to Polish conditions [11]. 

To reduce the consequences of COVID-19 infection, the consensus recommends integrated multidisciplinary rehabilitation services for individuals with long-term effects of COVID-19 [12]. It requires finding therapeutic solutions to fight against post-COVID-19 conditions to reach a new level of evidence-based medicine and improve the quality of survivors’ lives [1]. So far, training programs have already been used that only improve balance without considering the increase in muscle strength and improvement of the patient’s functional condition [13]. We know that long-term strength training improves muscle strength and physical functioning in older adults [14]. Moreover, resistance training (RT) has been shown to have a positive impact on body balance disorders in older people [14]. RT is a safe and effective method in combatting muscle mass and declining functional capacity in the elderly [15]. RT can improve muscle strength, which is a key factor for maintaining balance and preventing falls. Furthermore, RT has been compared to other types of training, such as pilates and multicomponent training, and has been found to be equally or more effective in improving balance and preventing falls [16].

Therefore, the aim of the above study was to create a specific, early, and safe protocol-based intervention to improve functional and equilibrium abilities in older adults impacted by COVID-19. We hypothesize that resistant training improves postural stability and quality of life in post-COVID-19 survivors.

## 2. Materials and Methods

### 2.1. Participants

The participants were recruited from various sources, including nursing homes, primary health care facilities, a University of the Third Age, social media of the local university, and surrounding communities. Inclusion criteria for the study were individuals of both sexes aged 65 and older, with a positive RT-PCR test and/or positive results in tests for antibodies against the SARS-CoV-2 coronavirus conducted 3–12 months prior to the study. Participants also needed to report one or more post-COVID-19 signs and symptoms, such as fatigue, muscle weakness, dizziness, headache, memory and concentration disorders, exercise intolerance, and depression. Before starting the program, participants underwent screening by a physician, and the exacerbation of post-exercise symptoms was assessed using a questionnaire [17] and an orthostatic test [18]. The exclusion criteria for the study included age under 65, active cardiac disease, oxygen desaturation below 95% for more than 1 min, dysfunctions of the autonomic nervous system (orthostatic intolerance), and serious health conditions such as cancer.

Importantly, 92% of respondents had been vaccinated with at least one dose of the anti-SARS-CoV-2 vaccine, and only 27% of study participants had become ill before vaccination. The mean time from onset of illness for those classified according to the inclusion criteria was 9 months, and 33% described the infection as mild, 51% as moderate, 10% as severe, and 6% as very severe. After meeting inclusion criteria and passing medical screening, participants were randomly allocated to either the intervention group, which received resistance training, or the control group, which was advised to maintain their usual activity level. Random allocation to groups was carried out using an Excel random number generator. Two members from the intervention group dropped out due to pain unrelated to participation in the exercise program (one due to low back pain and one due to knee pain). Additionally, three participants from the control group did not attend the post-test stage. In the end, a total of 46 participants successfully completed the study protocol, including both pre- and post-testing. The data from these 46 post-COVID-19 seniors were analyzed. On average, the time from the onset of the disease in individuals meeting the inclusion criteria was 9 months. Table 1 presents the anthropometric characteristics of the tested groups at baseline. There were no significant differences between the intervention and control groups in anthropometric parameters, except for age in the male groups (*p* < 0.05).

Ethical clearance for the study was granted by the local Ethics Committee (SKE 01-41/2022). The study protocol was registered on clinicaltrials.org (NCT05934279). All subjects provided written informed consent prior to data collection. The necessary minimum total number of subjects (n = 40) was obtained using the G*Power program, assuming the detection of medium-sized effects (η^2^ = 0.06) at a significance level of a = 0.05 and statistical power of 0.85. 

### 2.2. Postural Stability Evaluation

Stabilographic assessments were conducted to evaluate postural stability using the Biodex Balance System SD platform (USA) by Biodex (BBS). Three protocols, each lasting 20 s with 10 s breaks, were implemented on the BBS. This system allows subjects to undergo testing on a platform ranging from stable to unstable across 12 levels, with the degree of instability increasing from level 12 (most stable) to level 1. The Postural Stability Test (PST) was performed on a stationary platform with both eyes open (EO) and closed (EC). The test aimed to ascertain the overall stability index (OSI), anterior–posterior stability index (APSI), and medial–lateral stability index (MLSI). Additionally, the fall-risk test was conducted with EO on an unstable platform, varying the levels from 12 to 8 and from 6 to 2. This test facilitated the determination of the fall-risk index (FRI). High values of all these indices comprised the body balance disorders.

### 2.3. Quality of Life Assessment

The WHOQOL-BREF [10], an abbreviated 26-item version of the WHOQOL-100 (WHOQOL Group 1995, 1998) was used to assess the QOL [9,19]. It contains 1 general QOL item, 1 general health item, and 24 specific items that cover four domains: physical (7 questions included items on mobility, daily activities, functional capacity, energy, pain, and sleep), psychological (6 questions concerned self-image, negative thoughts, positive attitudes, self-esteem, mentality, learning ability, memory concentration, religion, and the mental status), social relations (3 questions on personal relationships, social support, and sex life), and environmental (8 questions covered issues related to financial resources, safety, health and social services, living physical environment, opportunities to acquire new skills and knowledge, recreation, general environment: noise, air pollution, etc., and transportation). Moreover, the scores from the first (Q1—How would you rate your quality of life?) and second (Q2—How satisfied are you with your health?) questions were taken into statistical analysis. The items were answered on five-point scales, which assess the intensity (nothing–extremely), capacity (nothing–completely), frequency (never–always), and evaluation of QOL facets (very dissatisfied–very satisfied; very bad–very good) with respect to the last two weeks. Negatively keyed items were reversely scored. The raw scores were then transformed linearly to a 0–100 scale. Domain scores are scaled in a positive direction (a higher score indicates a higher quality of life).

### 2.4. Intervention

Resistance training (RT) that focused on enhancing muscle strength was conducted twice a week, with each session lasting 60 min over an 8-week period, following the guidelines provided by World Physiotherapy and NICE [20]. Prior to each session, heart rate, blood pressure, and oxygen saturation were assessed. If blood pressure exceeded >160/100 mmHg, heart rate (HR) was >100 or <50 beats per minute, participants were not permitted to engage in exercises during that session.

During the first training session, participants underwent the determination of 1 Repetition Maximum (1RM) for each exercise. This involved 4–5 trials with increasing load, and rest periods between trials were set at 3 min of passive recovery. The objective was to complete 3–5 repetitions with the maximum load. Participants were instructed to perform the exercises at a comfortable pace. The 1RM was calculated using the formula developed by Brzycki [21].

**Figure 1:**
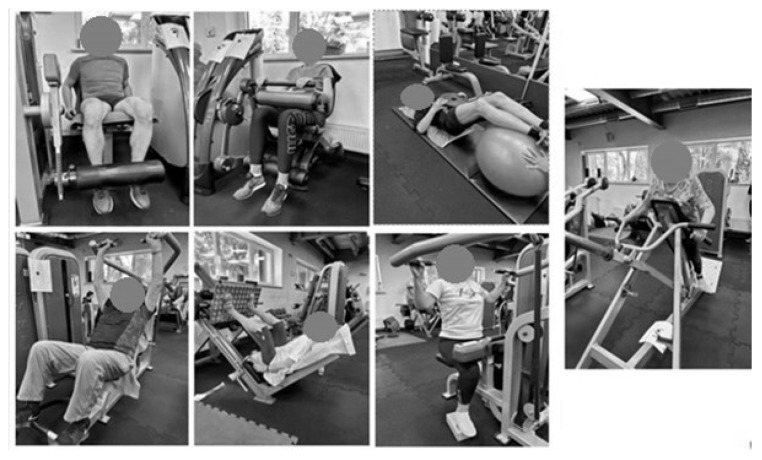
Examples of exercises in each session.

Each training session aimed to achieve an exercise intensity of 70% of 1RM and consisted of three sets of 12 repetitions for each exercise, including incline bench press, 45 degrees leg press, latissimus pull-down, trunk crunch, T-bar row, leg extension, and leg curl (Figure 1).

The rest periods between sets comprised a 2 min passive recovery. Prior to each training session, participants engaged in a 15 min general warm-up on an orbitrec or treadmill with individual intensity set at 60–65% of HRmax. The training loads were adjusted individually, increasing by 5 kg when a subject successfully completed all repetitions during an exercise.

### 2.5. Statistical Analysis

Statistical analyses were carried out using Statistica 14.0. The normality of the distributions of the study variables was assessed using the Shapiro–Wilk test. Since the variables tested do not meet the condition of normality of distributions, the Mann–Whitney U test was used for comparisons between groups. Changes in variables before and after the intervention were assessed using the Wilcoxon test. The response to the intervention was assessed by comparing the increments of the study variables in the two groups using the Mann–Whitney U test. Effect sizes were assessed by Glass’s rank-biserial correlation coefficient (Mann–Whitney test) and equivalent correlation coefficient (Wilcoxon test). A significance level of α = 0.05 was assumed. 

## 3. Results

The results of older adults in the intervention group who did not miss more than three sessions were taken for analysis. The average attendance rate was 93% (80–100%). The results of two testing sessions (pre- and post-test) of postural stability are shown in Table 2. At the baseline, the groups (control and intervention) did not differ significantly in any of the study variables. 

After the intervention, the statistical analysis revealed significant differences in OSI EO, APSI EO, and FRI 6-2 values in males (*p* < 0.05) and APSI EO (*p* < 0.05) values in females compared to control groups, respectively. In the training female group, a significant improvement was also reported in static postural stability parameters: OSI EO and APSI EO (*p* < 0.05) compared to baseline results. Moreover, in the dynamic conditions, the analysis revealed the improvement in FRI 6-2 values in the intervention group in both genders (*p* < 0.01 and *p* < 0.05 for men and women, respectively).

To assess the effect of intervention, the increments of the study variables of postural stability were analyzed. Greater improvement was recorded in the intervention group in the OSI EO (Z = −3.12, *p* < 0.01, R = 0.533) and the FRI 6-2 (Z = −2.06, *p* < 0.05, R = 0.354) (Table 2). 

Additionally, the significantly different reactions of the groups were observed in the psychological domain (DOM2) (Z = 2.194, *p* < 0.028, R = 0.389) and social relationship domain (DOM3) (Z = 2.051, *p* < 0.0403, R = 0.361), as well as in question 2 concerning general health (Z = 3.309, *p* < 0.0009, R = 0.535). The detailed results from the above analysis are shown in Table 3 (Table 3).

## 4. Discussion

Postural stability decreases with age, mainly due to a decrease in muscle mass and strength caused by changes in the nervous system and in the muscles themselves because of less involvement in physical activities. For this reason, the guidelines of many geriatric societies primarily recommend exercises in the form of resistance (strength), balance, gait, and coordination training, as they are effective in reducing the risk of falls [22]. In people after COVID-19, the ability to maintain balance is often limited due to general weakness and impaired function of the sensory organs. The balance deficit observed in people after COVID-19 leads to an impaired ability to perform typical daily activities [23]. 

The immune response to SARS-CoV-2 infection is usually characterized by a complex interplay between innate and adaptive immune mechanisms. The production of autoantibodies and immune complexes can further exacerbate tissue damage and inflammation. These processes can contribute to widespread inflammation and tissue damage and thus be associated with sensory organ dysfunction [24]. One of the consequences of COVID-19 after infection can be severe neuronal changes that impair the ability of the central nervous system to respond effectively to visual, vestibular, and proprioceptive postural feedback. Studies have shown that negative changes in the vestibular organs in people after COVID-19 infection can persist for months, and dizziness caused by the SARS-CoV-2 virus may be related to the involvement of the vestibular and visual systems [25].

In the above study, participants in the intervention group showed a significant improvement in OSI EO, APSI EO, and FRI 6-2 in men and APSI EO in women compared to the control groups. The training of the women led to a significant improvement in the static postural stability parameters (OSI EO and APSI EO) compared to the baseline values. However, only in the case of OSI EO can there be a large effect related to the improvement obtained in the intervention group compared to the control group. It should be emphasized that tests under static conditions (on a stationary platform) do not fully reflect the complexity of the balance control mechanism. To assess postural stability, a test should be performed not only on a static platform but also on an unstable surface. The analysis under dynamic conditions showed an improvement in the FRI 6-2 value in the intervention group for both genders. A comparison of these changes with those obtained in the control group showed that, in this case, the effect was moderate. The prepared training method aimed at increasing muscle strength proved to be effective in improving OSI EO in the intervention group, which also reflected a reduction in the risk of falls in this group. Based on the large effect size, it can be concluded that RT proved to be effective in preventing falls.

Falls are one of the main causes of reduced mobility and reduced quality of life in older people. There is evidence that estrogen deficiency is related to fall risk in women. Estrogen deficiency can contribute to muscle weakness and changes in muscle mass, which can affect overall strength and balance [26]. Estrogen receptors are present in tissues involved in proprioception, which is the body’s ability to sense its position in space, and estrogen deficiency can impair this sensory feedback [27]. Impaired proprioception can affect coordination and balance, making it more difficult for women to respond appropriately to changes in their environment and avoid falls. Moreover, women with estrogen deficiency may have difficulty with activities of daily living, increasing their susceptibility to falls.

The results of this study, which was conducted on a group of men and women over the age of 65, show that resistance exercises significantly improve postural stability parameters and reduce the risk of falls in both intervention groups. It is noteworthy that the men in the intervention group initially performed worse than the women in terms of FRI 6-2 fall risk (poorer balance) after the COVID-19 study and that the motor tasks they performed in the form of resistance training (RT) led to a significantly greater improvement in FRI 6-2 compared to the women. These results could be due to changes in the functioning of the systems regulating balance and ensuring postural stability after COVID-19. Research by Mustafa and Taya (2020) (2020) has shown that SARS-CoV-2 infections cause the occurrence of numerous vestibular disorders, such as vestibular neuritis, benign paroxysmal vertigo, and orthostatic dysfunction [28]. In postmenopausal women in the intervention group, the results confirmed that RT significantly reduces the risk of falls. 

Another aspect analyzed in the work was the study of the impact of RT in people after COVID-19 on quality of life. Physical activity (PA) has a strong, well-documented relationship with quality of life (QOL) dimensions such as physical health, psychological well-being, social relationships, and environment [29]. Therefore, our hypothesis that RT would improve people’s QOL after COVID-19 seemed justified.

These results confirm that regular exercise in older people over a longer period has a significant impact on elements of mental well-being and quality of life. People in the intervention group differed significantly in the degree of satisfaction with their health (Q2: How satisfied are you with your health?). Compared to the control group, they reported a higher level of satisfaction (despite several dysfunctions). This confirms that even a gradual functional improvement in physical and mental health, social relationships, and environment has a significant impact on mental and physical health and contributes to improving quality of life. These results confirm that an eight-week resistance training program led to significant differences between the intervention and control groups in terms of psyche, social relationships, and general health. 

The improvement in participants’ “DOM2 psychology” after the RT intervention included variables such as positive feelings, thinking, learning, memory and concentration, self-esteem, body image and appearance, negative feelings, spirituality/religion/personal beliefs. It is likely that RT could influence neurophysiological mechanisms, leading to increased cerebral blood flow and angiogenesis, which improves cognitive health [30]. Combined cognitive–motor training (CMT) enabled older adults to perform a cognitive task and balance exercises simultaneously. The simultaneous inclusion of motor and cognitive activities led to an improvement in mental and physical abilities, which in turn improved mental well-being and quality of life [31]. Collinet and Delalandre (2017) showed that performing strength tasks/exercises led to an increase in strength and energy, improved the ability to perform daily activities, and was associated with improved physical functioning, which in turn was reflected in better cognition in older people [32]. In turn, Kekaelaeinen et al. (2018) found that any type of CMT intervention had a positive effect on improving empathy and QOL symptoms, cognitive health, and social participation in older adults [33].

Regarding the studied variable “DOM3 social relationships” (personal relationships, social support, and sexual activity), the results of the above research showed that participation in RT significantly influences the improvement of the tested variables. During the pandemic, the frequency of social activities decreased significantly in both genders (while it was higher in older women than in older men before the lockdown). The results of a study conducted by Reher et al. (2020) showed a significant reduction in social activities, feelings of extreme isolation, and anxiety due to house arrest in older adults living alone [34]. Loneliness is an objective expression of isolation; therefore, there is an increased risk of social isolation due to a lack of contact opportunities and social networks [35]. There is ample evidence in the literature confirming the link between social isolation and health. Many studies have shown that social isolation is related to physical health, from immune responses (e.g., increased pro-inflammatory activity) to clinical responses (e.g., increased risk of coronary heart disease and stroke) [36,37]. In addition to physical health, social isolation can also have a negative impact on cognitive function, mental health, and health-related behaviors [38]. In addition, training for older people after COVID-19, conducted in a group format, ensures the need for contact with other “survivors” of the pandemic and thus fulfills psychosocial needs. Some authors emphasize that group training helps to reduce stress levels, increase enjoyment of exercise and self-confidence, and improve social skills [39]. 

Different data from the same group of patients were published earlier in the paper by Kaczmarczyk et al. [40].

### Limitation of the Study

The first limitation is the small number of subjects in the study. While larger numbers would be preferred, the size is appropriate considering that this is one of the first studies utilizing an active exercise program following the end of the public health emergency. It was important to establish the safety and effectiveness of the protocol before increasing the size of the cohort. The second limitation is that the participants in the intervention group were already functioning at a reasonably high level. Once again, establishing efficacy in a higher-functioning group was an important first step. It is likely that a group functioning at a lower level would have achieved even greater gains. The third limitation is that we were not able to systematically track symptom improvement within the control or intervention group. Future studies will expand on this work by using a larger cohort and assessing the impact on a cluster of symptoms. The last limitation is that we used a community gym equipped with resistance exercise machines. That resource may not be available in every community, and modifying the exercise program with body weight resistance may be worthwhile. If specialist equipment is not available, other types of resistance utilizing dumbells or resistance bands may yield similar results.

## 5. Conclusions

The resistance training protocol used in the above study had a positive effect on older adults affected by COVID-19 and led to a significant improvement in their postural stability. These results show that resistance exercises significantly improve postural stability parameters and reduce the risk of falls in both intervention groups. Furthermore, they confirmed that regular exercise of older people over a longer period has a significant impact on elements of psychological well-being and quality of life.

Application conclusion: Resistance exercises should be included as part of the rehabilitation/therapy process in the standard management of seniors with post-COVID-19 symptoms.

## Figures and Tables

**Table 1:** Anthropometric characteristics of the tested groups at baseline.

		n	Age [Years]	Body Mass [kg]	Body Height [cm]	BMI [kg/m^2^]
Intervention group	F	11	69.27 ± 5.20	65.27 ± 10.54	163.09 ± 7.61	24.50 ± 4.83
	M	15	69.47 ± 4.84 *	87.67 ± 15.10	176.67 ± 6.85	27.86 ± 3.38
Control group	F	12	73.33 ± 7.39	72.59 ± 12.13	160.92 ± 5.74	28.06 ± 3.34
	M	8	75.63 ± 7.07	89.45 ± 20.29	179.00 ± 6.12	27.07 ± 5.36

Legend: Data presented as mean (SD); F—females; M—males; BMI—body mass index; *—*p* < 0.05.

**Table 2:** The results of the postural stability evaluation.

		Control		Intervention		Increments (After–Before) Comparisons Control vs. Intervention		
		Male (n = 8)	Female (n = 12)	Male (n = 15)	Female (n = 11)			
		Median (LoQ-UpQ)	Median (LoQ-UpQ)	Median (LoQ-UpQ)	Median (LoQ-UpQ)	Z	*p*	R
OSI EO	Before	0.40 (0.30–0.45)	0.30 (0.30–0.40)	0.40 (0.30–0.50)	0.40 (0.30–0.40)	−3.12	0.0017 *	0.533
OSI EO II	After	0.45 (0.35–0.75)	0.30 (0.25–0.45)	0.30 (0.30–0.40) *	0.30 (0.20–0.30) ^#^			
APSI EO	Before	0.30 (0.25–0.40)	0.30 (0.20–0.30)	0.20 (0.20–0.30)	0.30 (0.20–0.30)	−1.67	0.1087	0.281
APSI EO II	After	0.30 (0.25–0.60)	0.20 (0.20–0.30)	0.20 (0.20–0.30) *	0.20 (0.10–0.20) *^,#^			
MLSI EO	Before	0.10 (0.10–0.20)	0.10 (0.10–0.20)	0.10 (0.10–0.20)	0.20 (0.10–0.30)	−1.83	0.0860	0.298
MLSI EO II	After	0.20 (0.15–0.30)	0.10 (0.10–0.25)	0.10 (0.10–0.20)	0.10 (0.10–0.20)			
OSI EC	Before	1.45 (1.05–1.50)	1.20 (0.80–1.40)	1.30 (0.80–1.60)	1.10 (0.80–1.50)	0.55	0.5755	−0.098
OSI EC II	After	1.20 (0.95–1.45)	0.95 (0.75–1.30)	1.10 (0.80–1.80)	1.00 (0.70–1.10)			
APSI EC	Before	0.85 (0.70–1.20)	0.70 (0.55–1.00)	1.00 (0.80–1.20)	0.80 (0.60–1.20)	−0.29	0.7671	0.052
APSI EC II	After	0.85 (0.70–1.25)	0.85 (0.65–1.10)	1.00 (0.70–1.60)	0.70 (0.60–0.90)			
MLSI EC	Before	0.60 (0.50–0.90)	0.50 (0.40–0.80)	0.40 (0.30–0.80)	0.50 (0.20–0.70)	0.71	0.4752	−0.125
MLSI EC II	After	0.55 (0.40–0.75)	0.30 (0.20–0.60)	0.30 (0.20–0.50)	0.40 (0.20–0.50)			
FRI 12-8	Before	1.20 (1.00–1.75)	0.85 (0.80–1.10)	1.10 (0.90–1.40)	1.10 (0.70–1.20)	−0.57	0.5755	0.100
FRI12-8 II	After	1.10 (1.00–1.80)	1.00 (0.75–1.15)	1.10 (1.00–1.20)	0.80 (0.70–1.00)			
FRI 6-2	Before	10.00 (7.70–10.00)	2.20 (1.45–10.00)	6.20 (2.70–10.00)	2.20 (1.20–4.50)	−2.06	0.0418 *	0.354

Notes: *—different than control; ^#^—difference before–after; OSI—overall stability index; APSI—anterior–posterior stability index, MLSI—medial–lateral stability index, FRI—fall-risk index at various levels ranges: 12-8 and 6-2; EO—eyes open; EC—eyes closed; *,^#^—*p* < 0.05.

**Table 3:** The results of the quality-of-life evaluation.

		Control		Intervention		Increments (After–Before) Comparisons Control vs. Intervention		
		Male (n = 8)	Female (n = 12)	Male (n = 15)	Female (n = 11)	Z	*p*-Value	R
		Mean, Me (LoQ-UpQ)	Mean, Me (LoQ-UpQ)	Mean, Me (LoQ-UpQ)	Mean, Me (LoQ-UpQ)			
DOM1 Physic.	B	57.3, 59.5 (47.0–66.0)	50.2, 53.0 (38.0–56.0)	54.3, 56.0 (44.0–63.0)	57.5, 56.0 (44.0–69.0)	0.412	0.6804	0.075
DOM1 Physic.	A	51.8, 50.0 (44.0–59.5)	57.3, 56.0 (56.0–56.0)	59.0, 63.0 (50.0–69.0)	59.3, 63.0 (56.0–63.0)			
DOM 2 Psychol.	B	67.3, 66.0 (59.5–75.0)	59.5, 59.5 (56.0–66.0)	62.6, 63.0 (56.0–69.0)	64.4, 69.0 (56.0–69.0)	2.194	0.0282 *	0.389
DOM 2 Psychol.	A	60.3, 59.5 (56.0–63.0)	61.4, 63.0 (56.0–69.0)	69.7, 69.0 (63.0–81.0)	68.4, 69.0 (63.0–69.0)			
DOM3 Soc.	B	66.4, 62.5 (53.0–78.0)	64.5, 69.0 (56.0–75.0)	67.1, 75.0 (56.0–75.0)	68.7, 69.0 (56.0–81.0)	2.051	0.0403 *	0.361
DOM3 Soc.	A	68.0, 62.5 (56.0–84.5)	62.5, 72.0 (50.0–75.0)	72.1, 75.0 (69.0–75.0)	79.0, 75.0 (69.0–81.0)			
DOM4 Environ.	B	74.3, 75.0 (62.5–84.5)	68.8, 69.0 (69.0–75.0)	71.1, 69.0 (63.0–75.0)	74.6, 75.0 (69.0–81.0)	0.706	0.4802	0.126
DOM4 Environ.	A	75.1, 78.0 (66.0–81.0)	72.0, 72.0 (63.0–81.0)	78.1, 75.0 (69.0–88.0)	77.9, 81.0 (69.0–88.0)			
Q1	B	4.1, 4.0 (4.0–4.5)	3.8, 4.0 (3.0–4.5)	3.8, 4.0 (3.0–4.0)	4.0, 4.0 (4.0–4.0)	1.175	0.2399	0.181
Q1	A	4.1, 4.0 (4.0–4.0)	3.9, 4.0 (4.0–4.0)	4.2, 4.0 (4.0–5.0)	4.2, 4.0 (4.0–4.0)			
Q2	B	3.4, 3.5 (2.5–4.0)	3.3, 3.5 (2.5–4.0)	3.1, 3.0 (2.0–4.0)	3.5, 4.0 (3.0–4.0)	3.309	0.0009	0.535
Q2	A	3.0, 3.0 (2.0–4.0)	3.3, 3.0 (3.0–4.0)	3.9, 4.0 (4.0–4.0)	3.8, 4.0 (4.0–4.0)			

Notes: DOM1 Physic.—domain 1: physical health; DOM2 Psychol.—domain 2: psychological; DOM3 Soc.—domain 3: social relationships; DOM4 Environ.—domain 4: environment; Q1—How would you rate your quality of life?; Q2—How satisfied are you with your health?; score ≤ 45—low QOL; score 46–65—moderate QOL; score > 65—relatively high QoL; B—before; A—after; *—*p* < 0.05.

## Data Availability

The datasets generated and analyzed during the current study are not publicly available due to the restrictions involved when obtaining ethical approval for this study, which commit us to only share the data with members of the research team but allow data to be made available from the corresponding author upon reasonable request.

## References

[1] Carley S., Horner D., Body R., Mackway-Jones K. (2020). Evidence-based medicine and COVID-19: What to believe and when to change. Emerg. Med. J..

[2] Centers for Disease Control and Prevention (2023). Long COVID or Post-COVID Conditions.

[3] Pazdro-Zastawny K., Dorobisz K., Misiak P., Kruk-Krzemień A., Zatoński T. (2022). Vestibular disorders in patients after COVID-19 infection. Front. Neurol..

[4] Almufarrij I., Uus K., Munro K.J. (2020). Does coronavirus affect the audio-vestibular system? A rapid systematic review. Int. J. Audiol..

[5] Viola P., Ralli M., Pisani D., Malanga D., Sculco D., Messina L., Laria C., Aragona T., Leopardi G., Ursini F. (2021). Tinnitus and equilibrium disorders in COVID-19 patients: Preliminary results. Eur. Arch. Otorhinolaryngol..

[6] Bobowik P., Wiszomirska I., Gajewski J., Kaczmarczyk K. (2023). Muscle strength and equilibrium-maintaining ability in post-COVID women. Gait Posture.

[7] Dzięcioł-Anikiej Z., Dakowicz A., Dzięcioł J., Kopko S., Moskal-Jasińska D., Gawlikowska-Sroka A., Kuryliszyn-Moskal A., Kostro A.M. (2023). Balance Disorders in People with History of COVID-19 in Light of Posturographic Tests. J. Clin. Med..

[8] Rosa M., Graça M.C., Duarte M., Martins N., Sanches L., Silva E., Ferreira L., Seixas A. (2023). Barriers, facilitators, and impact of the COVID-19 pandemic on the physiotherapy intervention for people with dementia or cognitive impairment. Adv. Rehabil..

[9] (1998). The World Health Organization Quality of Life Assessment (WHOQOL): Development and general psychometric properties. Soc. Sci. Med..

[10] Skevington S.M., Lotfy M., O’Connell K.A., WHOQOL Group (2004). The World Health Organization’s WHOQOL-BREF quality of life assessment: Psychometric properties and results of the international field trial. Qual. Life Res..

[11] Wołowicka L., Jaracz K. (1998). Health-related quality of life in self-reported surveys. Adv. Nurs. Health Promot..

[12] Maley J.H., Alba G.A., Barry J.T., Bartels M.N., Fleming T.K., Oleson C.V., Rydberg L., Sampsel S., Silver J.K., Sipes S. (2022). Multi-disciplinary collaborative consensus guidance statement on the assessment and treatment of breathing discomfort and respiratory sequelae in patients with post-acute sequelae of SARS-CoV-2 infection (PASC). PM R..

[13] Jalilzadeh Afshar P. (2021). Vestibular Rehabilitation in Isolated Otolith Dysfunction after Covid-19: A Case Report. Iran. Rehabil. J..

[14] Aartolahti E., Lönnroos E., Hartikainen S., Häkkinen A. (2020). Long-term strength and balance training in prevention of decline in muscle strength and mobility in older adults. Aging Clin. Exp. Res..

[15] Endo Y., Nourmahnad A., Sinha I. (2020). Optimizing Skeletal Muscle Anabolic Response to Resistance Training in Aging. Front. Physiol..

[16] Sousa N., Mendes R. (2015). Comparison of effects of resistance and multicomponent training on falls prevention in institutionalized elderly women. J. Am. Geriatr. Soc..

[17] Cotler J., Holtzman C., Dudun C., Jason L.A. (2018). A Brief Questionnaire to Assess Post-Exertional Malaise. Diagnostics.

[18] Freeman R., Wieling W., Axelrod F.B., Benditt D.G., Benarroch E., Biaggioni I., Cheshire W.P., Chelimsky T., Cortelli P., Gibbons C.H. (2011). Consensus statement on the definition of orthostatic hypotension, neurally mediated syncope and the postural tachycardia syndrome. Clin. Auton. Res..

[19] (1995). The World Health Organization Quality of Life assessment (WHOQOL): Position paper from the World Health Organization. Soc. Sci. Med..

[20] NICE (2020). COVID-19 Rapid Guideline: Managing the Long-Term Effects of COVID-19. https://www.nice.org.uk/guidance/ng188.

[21] Brzycki M. (1993). Strength testing—Predicting a one-rep max from reps to fatigue. J. Phys. Educ. Recreat. Danc..

[22] Panel on Prevention of Falls in Older Persons, American Geriatrics Society and British Geriatrics Society (2011). Summary of the Updated American Geriatrics Society/British Geriatrics Society clinical practice guideline for prevention of falls in older persons. J. Am. Geriatr. Soc..

[23] Mandal S., Barnett J., Brill S.E., Brown J.S., Denneny E.K., Hare S.S., Heightman M., Hillman T.E., Jacob J., Jarvis H.C. (2021). Long-COVID: A cross-sectional study of persisting symptoms, biomarker and imaging abnormalities following hospitalisation for COVID-19. Thorax.

[24] Xu E., Xie Y., Al-Aly Z. (2022). Long-term neurologic outcomes of COVID-19. Nat. Med..

[25] Harapan B.N., Yoo H.J. (2021). Neurological symptoms, manifestations, and complications associated with severe acute respiratory syndrome coronavirus 2 (SARS-CoV-2) and coronavirus disease 19 (COVID-19). J. Neurol..

[26] Collins B.C., Laakkonen E.K., Lowe D.A. (2019). Aging of the musculoskeletal system: How the loss of estrogen impacts muscle strength. Bone.

[27] Yang L., Xu Y., Zhang Y., Vijayakumar S., Jones S.M., Lundberg Y.W. (2018). Mechanism Underlying the Effects of Estrogen Deficiency on Otoconia. J. Assoc. Res. Otolaryngol..

[28] Mustafa M., Taya U. (2020). Vestibular Evoked Myogenic Potentials of Asymptomatic COVID-19 PCR-Positive Cases. Glob. J. Otolaryngol..

[29] Trabelsi K., Ammar A., Masmoudi L., Boukhris O., Chtourou H., Bouaziz B., Brach M., Bentlage E., How D., Ahmed M. (2021). Sleep Quality and Physical Activity as Predictors of Mental Wellbeing Variance in Older Adults during COVID-19 Lockdown: ECLB COVID-19 International Online Survey. Int. J. Environ. Res. Public Health.

[30] Rhyu I.J., Bytheway J.A., Kohler S.J., Lange H., Lee K.J., Boklewski J., McCormick K., Williams N.I., Stanton G.B., Greenough W.T. (2010). Effects of aerobic exercise training on cognitive function and cortical vascularity in monkeys. Neuroscience.

[31] Cao Z.-B., Maeda A., Shima N., Kurata H., Nishizono H. (2007). Effects of exercise and nutritional intervention to improve physical factors associated with fracture risk in middle-aged and older women. Int. J. Sport Health Sci..

[32] Collinet C., Delalandre M. (2017). Physical and sports activities, and healthy and active ageing: Establishing a frame of reference for public action. Int. Rev. Sociol. Sport.

[33] Kekalainen T., Kokko K., Sipila S., Walker S. (2018). Effects of a 9-month resistance training intervention on quality of life, sense of coherence, and depressive symptoms in older adults: Randomized controlled trial. Qual. Life Res..

[34] Reher D.S., Requena M., de Santis G., Esteve A., Livi Bacci M., Padyab M., Sandström G. (2020). The COVID-19 pandemic in an aging world. SocArXiv.

[35] National Academies of Sciences, Engineering, and Medicine, Division of Behavioral and Social Sciences and Education, Health and Medicine Division, Board on Behavioral, Cognitive, and Sensory Sciences, Board on Health Sciences Policy, Committee on the Health and Medical Dimensions of Social Isolation and Loneliness in Older Adults (2020). Social Isolation and Loneliness in Older Adults: Opportunities for the Health Care System.

[36] Valtorta N.K., Kanaan M., Gilbody S., Ronzi S., Hanratty B. (2016). Loneliness and social isolation as risk factors for coronary heart disease and stroke: Systematic review and meta-analysis of longitudinal observational studies. Heart.

[37] Smith K.J., Victor C. (2019). Typologies of loneliness, living alone and social isolation, and their associations with physical and mental health. Ageing Soc..

[38] Floyd A., Moyer A. (2009). Group vs. individual exercise interventions for women with breast cancer: A meta-analysis. Health Psychol. Rev..

[39] Zhu L., Jiang X., Sun Y., Shu W. (2016). Effect of hormone therapy on the risk of bone fractures: A systematic review and meta-analysis of randomized controlled trials. Menopause.

[40] Kaczmarczyk K., Matharu Y., Bobowik P., Gajewski J., Maciejewska-Skrendo A., Kulig K. (2024). Resistance Exercise Program Is Feasible and Effective in Improving Functional Strength in Post-COVID Survivors. J. Clin. Med..

